# Global Health Security Risk Assessment in the Biological Threat Reduction Program

**DOI:** 10.1089/hs.2019.0132

**Published:** 2020-06-17

**Authors:** Nino Kharaishvili, Toni-Marie L. Hudson, Jaya K. Kannan, Vera Ettenger, Seema Mirje

**Affiliations:** Nino Kharaishvili, MD, MBA, is Principal, Health System Resilience, Federal and Environmental Solutions, Jacobs, Arlington, VA.; Toni-Marie L. Hudson, MSPH, and Seema Mirje, MD, are Associates; Vera Ettenger, PhD, is a Lead Associate; all at the Global Defense Group, Booz Allen Hamilton, McLean, VA.; Jaya K. Kannan, DVM, is a Veterinary Epidemiologist, Defense Systems, Northrop Grumman Corporation, McLean, VA.

**Keywords:** Healthcare system assessment, Global health security, Biological Threat Reduction Program, Public health preparedness/response, Surveillance

## Abstract

In 2014, the Biological Threat Reduction Program (BTRP) developed a country assessment tool to assess the risk to a country's biosurveillance, biosafety, and biosecurity systems and their vulnerability to naturally occurring, accidental, or nefarious release of weaponizable pathogens. The country assessment tool is a unique method of assessing public health and veterinary systems at the national and subnational levels. The assessment process is led by a multisectoral, multidisciplinary team composed of 8 subject matter experts who conduct a combination of document reviews, individual and focus group interviews, and in-person assessments. The intent of the tool was to standardize the BTRP program planning process and support quantitative metrics to measure partner country capacities and capabilities throughout BTRP engagement. Used in more than 25 countries to establish a baseline of the health security risk landscape, the tool provides a foundation for identifying and prioritizing system-wide risk mitigation and management activities as well as periodic evaluations of the impacts of these activities.

## Introduction

The United States Department of Defense plays a significant role in the US government in addressing infectious disease threats—whether from natural disease occurrences or accidental or intentional release of pathogens, in support of global health security—defined as “activities required to minimize the danger and impact of acute health events that endanger the collective health of populations living across geographical regions and international boundaries.”^1,2^ The US national security strategy specifically identifies preventing, detecting, and reporting disease outbreaks in real-time and responding more rapidly and effectively as a cornerstone of national security.^3^ One of the Department of Defense programs that contributes to global health security, is the Cooperative Threat Reduction's Biological Threat Reduction Program (BTRP) under the Defense Threat Reduction Agency. As part of its mission, BTRP addresses health security risks by working with US government partners and international organizations to promote international standards, regulations, and guidelines related to countering biological threats and weapons. The program has cooperative engagements with partner nations around the world to strengthen their ability to detect, diagnose, and report human, animal, and zoonotic disease cases and events.^4^ BTRP focuses on strengthening biosurveillance* (BSV) and biosafety^†^ and biosecurity^‡^ (BS&S) capabilities that address risks associated with select agents and toxins^§^ and emerging disease threats. In order to build these capabilities, the program develops risk management strategies in collaboration with partner nations, enabling them to detect, report, and respond to disease events in a timely fashion.

Although the BTRP's original mission was to counter weapons of mass destruction after the fall of the Soviet Union,^6,7^ the program has evolved to counter risks associated with emerging disease threats in partner nations across Africa, Europe, the Middle East, and Southeast Asia as well as risks from accidental or nefarious release of pathogens by state and nonstate actors. The evolution of BTRP has aligned with the growing global awareness of the threat of emerging and reemerging diseases and the importance of risk assessment and management to counter these threats.

Since the 1970s, newly emerging diseases have been identified at an unprecedented rate of 1 or more per year. Nearly 70% of these diseases are zoonotic, whose control may require surveillance of both human and animal health.^8^ As such, countries face new challenges in protecting their population from new and reemerging infectious diseases that can have devastating impact on their economy and future development.

The outbreak of severe acute respiratory syndrome (SARS) in 2003, in particular, awakened the world to threats ranging from newly emerging diseases to bioterrorism.^9^ The global impact of SARS led to the revision of the World Health Organization (WHO) International Health Regulations (IHR) in 2005 to address more modern threats.^10^ Yet, nearly 15 years after IHR (2005) entered into force, fewer than one-third of WHO member states meet the minimum requirements for core capacities needed to implement the regulations.^11^ Similarly, outbreaks of highly pathogenic avian influenza alerted human and animal health stakeholders to the risks of transboundary disease spread and pressured them to take steps toward managing crises at hand.^12,13^ These individual activities in the human, veterinary, and cross-sectoral domains are pieces of a global risk management framework driven, in part, by high-consequence pathogens. BTRP recognizes that achieving the capacities and capabilities to safely and securely detect, diagnose, and report endemic diseases can be challenging and supports countries in establishing disease baselines and routine practices that will contribute to rapidly detecting anomalous biological events.

Due to the wide range of possible risks across the program's partner nations and the varying capacities and capabilities among partners, BTRP begins its country engagements by conducting standardized in-depth health security risk assessments to identify constraints and opportunities for designing and implementing effective strategies for strengthening countries' health security preparedness and response capacity. Initiating country engagements with a health security risk assessment is integral to BTRP and necessary for identifying partner nations' priorities and appropriate risk mitigation strategies. Moreover, a systematic risk management approach is crucial for producing results that are consistent across BTRP and allows the program to use resources in an effective and efficient manner while furthering global health security.

## Rationale and Methodology

Prior to 2014, BTRP deployed a variety of disparate tools and processes for country assessments, which were unsuccessful in producing a comprehensive, repeatable, data-driven approach. Faced with an acute need for a more consistent, effective, and efficient methodology to understand countries' public health and veterinary system risks, the program conducted a thorough review of existing tools and guidance but did not find a standardized approach for assessing human and animal health threats or cross-cutting zoonotic domains.

In response, BTRP began to develop a comprehensive, modular, and rigorous approach for conducting country baseline assessments and subsequent periodic evaluations linked to semiquantitative metrics, which would be integrated into a strategic risk management plan. The tool needed to be based on international guidelines and best practices, tailored to the BTRP mission, and integrated with program objectives and existing planning cycles. In addition, BTRP wanted a standardized methodology that would produce consistent and repeatable results to establish a country's baseline, identify the next steps in a collaborative engagement, and measure each country's progress toward achieving mutually established goals or end states that comply with international requirements. The use of a modular approach, in particular, supported program goals to conduct periodic evaluations that focus on bilateral engagement areas (eg, if BTRP and a partner country focused their efforts on the biosecurity and biosafety domains, those sections of the assessment could be extracted and used to assess this area of engagement in the subsequent periodic evaluation). Lastly, the program wanted to develop an approach that would not only minimize in-country presence to the extent practicable but also be sensitive to “country assessment fatigue” in partner nations and save money and time.

When the country assessment tool was initially developed, the primary guiding documents for understanding and assessing BSV capacities included the WHO *Laboratory Assessment Tool*,^14^ World Organisation for Animal Health performance of veterinary services evaluation toolkit,^15^ IHR national legislation implementation tool,^16^ and IHR monitoring checklist.^17^ In addition, BTRP referenced a range of independently developed tools created in support of disease- or sector-specific projects sponsored by individual donor organizations such as the United States Agency for International Development and the US Centers for Disease Control and Prevention. As for the BS&S guiding documents, *Biosafety in Microbiological and Biomedical Laboratories*^18^ detailed acceptable biosafety practices specifically for the United States and has been widely adapted by multiple countries; the select agent section was particularly relevant as a reference. This section covers the characteristics and subsequent regulatory, safety, and security requirements for handling select agents. Although these guidelines and tools covered BSV or BS&S practices, no single consolidated approach captured all the guidelines for conducting a systematic assessment. To address the challenge in assessing health security risks in partner nations, BTRP developed a tailored approach that incorporated many of these references and practices into a single, defined methodology.

BTRP assembled a team of subject matter experts (SMEs) with expertise in various disciplines and domains, including human and veterinary clinical practice, epidemiology, laboratory sciences, health information technology, biosafety, biosecurity, social sciences, and monitoring and evaluation. The team began by first classifying healthcare systems risk drivers into categories, then determining that the country assessment tool would address 2 types of risks: those that BTRP could address and those that, while significant, could not be addressed because they are outside of the program's scope and mission. Table 1 outlines this initial breakdown.

**Table 1.** Risk Categories in the Biological Threat Reduction Program's Mission Space

Risk Categories	Examples
Risks that can be addressed as part of the Biological Threat Reduction Program's mission execution	Lack of/outdated policies and legislation that support risk identification and management (eg, no national disease surveillance list) Limited health professional training (eg, insufficient academic and in-service training for various health professionals) Insufficient biosurveillance infrastructure (eg, lack of information technology for electronic reporting, antiquated laboratory equipment for rapid disease identification) Insufficient biosafety and biosecurity infrastructure (eg, lack of biosafety cabinets and personal protective equipment, minimal security measures for pathogen repositories) Lack of/outdated health system standards and guidelines (eg, outdated laboratory diagnostic algorithms, clinical guidelines, case definitions)
Risks that cannot be directly addressed by the Biological Threat Reduction Program, although they affect implementation and sustainability of intervention activities	Critical infrastructure gaps (eg, lack of electrical supply for laboratories, inadequate transportation infrastructure) Human resource limitations (eg, human resource migration or “brain drain,” lack of funding for critical positions within health systems organizational structure) Inadequate resources for biological event response (eg, lack of vaccines or countermeasure stockpiles)

Following risk categorization, the team developed primary and supportive questions for assessing each individual risk driver. The questions were country agnostic, allowed for comprehensive data collection, and addressed all BTRP-specific lines of effort. SMEs presented their recommended questions to an internal peer-review panel for final adjudication, with question development and refinement taking multiple iterations until the team finalized the questionnaire.

After development, BTRP researched the most effective and efficient approach to implement the questionnaire. The goal was to collect a standardized and unbiased set of data quickly without sending a large contingent of SMEs to a country for an extended period. The team examined various implementation routes, including external and self-evaluation approaches and determined that implementing each approach individually would not meet the goal. BTRP concluded that exclusively using a country self-evaluation would introduce bias, but that a purely external evaluation by a team of SMEs would be cost prohibitive. As a result, the team developed a blended approach combining self-evaluation with external validation in which most of the work (described in the Results section) takes place outside of a country ahead of a relatively brief in-country visit. The team determined that this approach would reduce the resources required for conducting the risk assessment and introduce additional validity into findings, resulting in a higher quality, more cost-effective assessment.

## Results

After a research and piloting process, BTRP finalized the country assessment tool that included the BSV/BS&S assessment questionnaire and provided the implementation approach guided by quality assurance and quality control procedures.

### Assessment Questionnaire Description

The BSV/BS&S assessment questionnaire is a comprehensive and country-agnostic set of questions used to guide data collection by drawing information from documents, individuals, and other sources. Composed of nearly 1,000 questions and subquestions, the questionnaire is divided into domain- and discipline-specific sections and supports the research of risk categories outlined in the methodology section. The questions initiate data collection because they require teams to review documents, interview stakeholders, or solicit information through written correspondence.

The beginning of the questionnaire focuses on the contextual factors—demographics, politics, economy, threats (eg, criminal, terrorist), and vulnerabilities—that affect the operational environment and determinants that, in turn, impact health security. Although these factors cannot be directly influenced by BTRP, they may affect program implementation and activity sustainability in country (Table 1). Table 2 outlines contextual factors relevant to the BTRP mission execution.

**Table 2.** Biosurveillance/Biosafety and Biosecurity Assessment Questionnaire Contextual Factors

Area	Description
Major stakeholders and their area of responsibility	Mapping local and international institutes or agencies involved in human and animal health-related activities and their strategic plans for engagement, mission space, and overall progress made within partner country
National legislative and regulatory frameworks	Presence and level of implementation of any laws, rules, and/or regulations that affect the national human or animal health environment (eg, public health laws, mandated disease reporting, biosafety and biosecurity regulations and guidelines, laboratory rules of operation, health information sharing) as well as identification of key stakeholders involved in regulation, oversight, or performance of activities governed by human and animal health-related laws or policy
Budget and funding mechanisms	Overview of health systems funding and of funds allocated for maintenance and sustainment of specific health system components
Health system priorities	List of partner country priorities, particularly those related to infectious diseases; identification of healthcare burden that drives health project prioritization
International standards compliance	Description of how a partner nation meets or is working to meet international standards

The assessment questionnaire helps SMEs to understand the risks associated with each BTRP discipline (Table 3) and devotes a significant number of questions to investigating challenges in the public health and veterinary systems, using a One Health approach. The majority of questions were developed by BTRP in-house SMEs. Table 4 highlights an in-house developed question set.

**Table 3.** Biological Threat Reduction Program Disciplines in the Assessment Questionnaire

Disciplines	Profile of Disciplines
Human clinical Veterinary clinical Infectious disease epidemiology Laboratory science Health information technology Biosafety Biosecurity	Overview of the human and animal health systems as they relate to the biosurveillance and biosafety and biosecurity environment. This includes identification of relevant stakeholders and facilities and health priorities; descriptions of existing healthcare provider systems, disease surveillance/reporting systems, information technology systems supporting national health activities, and biosafety, biosecurity, and laboratory networks; and collaboration and communication with in-country health stakeholders. Biosurveillance and biosafety and biosecurity pillars focus on: • Roles and responsibilities • Infrastructure • Human resources and human resource development • Existing challenges or capacity gaps
One Health^a^	Existing intersectoral collaboration and communication, particularly between human and animal health stakeholders. This includes the structure and composition of multidisciplinary investigation teams and integration of biosafety and biosecurity components into the response.

^a^ “One Health” is not a discipline specific to Biological Threat Reduction Program but describes the intersection of the program's 7 disciplines.

**Table 4.** In-House Developed Question Set on Especially Dangerous Pathogen Consolidation and Security

Especially Dangerous Pathogen Consolidation and Security
1. Are there EDP repositories in the country? What pathogens, other than EDPs, are stored in national repositories? Are there known pathogen repositories outside of the national laboratory system? Are there guidelines or regulations specifying where pathogens are to be stored? Has a safe and secure method for pathogen storage (ie, cryopreservation) been identified and implemented in repositories?
2. Does the country actively promote pathogen consolidation and utilization of regional reference laboratories?
3. Are facilities actively conducting research using EDPs registered, inspected, and audited? Are there regulations for transfer of pathogens into and out of the facility?
4. Are there appropriate biosecurity measures in place for facilities that store EDPs? What physical security measures do they include (eg, access controls, intrusion detection, guard forces)? Is an up-to-date EDP inventory established and maintained? Is access to sensitive information (eg, inventory of pathogens) controlled by adequate policies and procedures? Are procedures for the safe and secure transport of culture, specimens, samples, and other biohazardous materials established and followed? If so, what are they? If not, what methods are currently used? What type of packaging is used? Has a risk assessment been conducted on shipping and receiving chains within the laboratory network in order to assure that sample risks conform to the destination facility biosafety level? Is there a mechanism to determine the personnel authorized to access EDPs?
5. What is the overall security posture of the country? Is the government stable? Are there active terrorist groups in the country/region? What types of criminal activities take place in the country?
6. Do facilities have integrated security programs? Do facilities utilize a threat assessment/vulnerability analysis approach to their security programs? Describe security measures employed at the facilities.

Abbreviation: EDP, especially dangerous pathogen.

### Implementation Description

**Figure 1.**
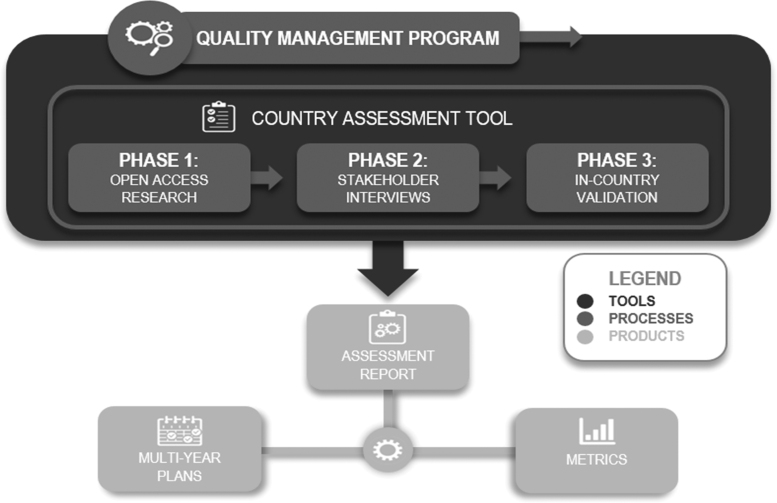
3-Phase Implementation Approach.

**Figure 2.**
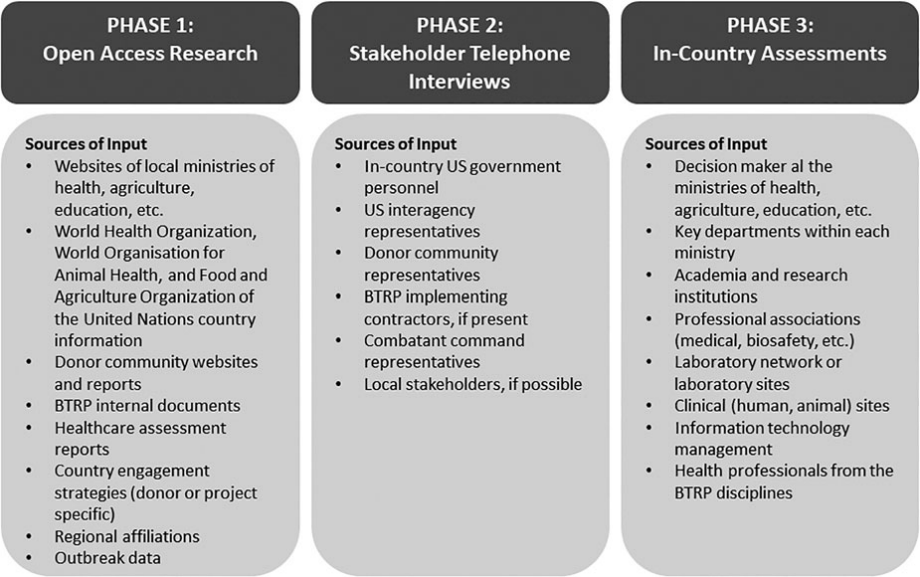
Sources of Information for the 3-Phase Implementation Approach. Abbreviation: BTRP, Biological Threat Reduction Program.

To reduce the high cost of human and financial resources associated with assessing each country, the team designed a 3-phased approach to standardize and streamline information collection and analysis (Figure 1). Figure 2 details sources of information that can be used to collect the information for each phase. While the list is not exhaustive, it provides a starting point for the assessment team. The process is managed by a robust quality assurance and control program that ensures high fidelity of data through a tiered approach: each phase validating and/or improving on the previous. This approach facilitates rapid appraisal and cost reduction and is composed of:

- Phase 1: Open Access Research. Open access research uses published literature, BTRP internal documents, and stakeholder websites to collect initial information to complete the BSV/BS&S assessment questionnaire. This step is conducted outside of an assessed country and may take up to 4 weeks to complete. Based on their research, SMEs also generate a list of key stakeholders to interview in subsequent phases.
- Phase 2: Telephone Interviews with Key Stakeholders. The assessment is further solidified through telephone interviews with US, international, and local country stakeholders identified during Phase 1. These interviews typically occur over the course of 4 weeks and are used to confirm Phase 1 findings and uncover information not available via open access research. After Phase 2, the assessment team obtains a more realistic understanding of the partner country BSV and BS&S systems' existing capabilities.
- Phase 3: In-Country Validation. SMEs travel to the country for up to 2 weeks to validate results found in the prior 2 phases, gain deeper insight into gaps and challenges, and gather more reliable data on the functioning and interaction of system components. Phase 3 is conducted via site visits, interviews with in-country stakeholders who were not previously interviewed during Phase 2, and focus groups. This phase may be supplemented with a hypothetical case scenario, such as a tabletop exercise.

Full implementation of all phases and the writing of the final assessment report requires a dedicated team of 8 to 12 SMEs and 10 to 14 weeks for completion. Typically, BTRP has relied on the team composition outlined in Table 5 for a standard country assessment. Depending on country size, scope of assessment, and duration allocated, the team may need to be augmented with other supportive SMEs if the standard approach is amended to cover additional components of the health system assessment.

**Table 5.** Suggested Composition of the Biological Threat Reduction Program Assessment Team

Primary Subject Matter Experts	Supportive Subject Matter Experts
Assessment team lead Medical specialist Veterinary specialist Epidemiology specialist Laboratory diagnostics specialist Information technology specialist Biosafety specialist Biosecurity specialist	General analyst Engineer Design architect Behavioral scientist

The final assessment report contains an analysis of a country's capacities, capabilities, and health security risks. Gaps and challenges identified during the assessment process are translated into recommendations, then incorporated into multiyear country engagement plans and used to develop baseline programmatic metrics to track BTRP's impact on achieving mutually established goals and end states in each partner country (Figure 1). The report is shared with the partner country and relevant US government organizations to facilitate future collaboration and support. Since this is a bilateral partnership, the report is not shared with a third country or international partners without permission from the assessed partner country.

### Country Assessment in Action

The BTRP assessment tool has been used in more than 25 countries in Africa, Europe, the Middle East, and Southeast Asia. Participating countries underwent varying levels of assessment, in which some completed only Phases 1 and 2 while others finished all 3 phases. The assessments were initiated by identifying a group of SMEs familiar with the BTRP assessment tool and mission. The SMEs completed Phase 1 by reviewing documents including internal BTRP material and open source documents indicated in Figure 2. On average, 250 documents were collected and analyzed for each country during the Phase 1 process.

The number of Phase 2 interviews with US, international, and in-country organizations ranged from 20 to 40. These interviews further validated findings from Phase 1 and obtained information for topics where open source research did not yield adequate results for the risk assessment.

During Phase 3, SMEs visited between 17 and 30 sites while in-country and conducted up to 150 stakeholder interviews across various regions in each country. Selected sites included local, regional, and national geographical locations and provided insight into capabilities at each level. The teams used focus groups, individual interviews, and direct observation, where applicable, to collect information.

SMEs used a standardized data collection form throughout all assessment phases to manage and validate findings and resources, allowing the assessor to build on knowledge from one phase to the next. The form also facilitated assessor analysis at the end of each phase and informed tailored and targeted questions in subsequent phases.

The final assessment reports were divided into 3 main sections: human health, animal health, and One Health. Within the human and animal health sections, the report provided summaries of stakeholders, national legislative and regulatory frameworks, budget and funding mechanisms, health system priorities, and international standards compliance as well as an overall health systems' overview. Subsections in the health systems component were structured along BTRP programmatic areas and outlined existing capacities, capabilities, system gaps, and recommendations to address gaps. Examples of a nonexhaustive list of pathogen consolidation and security gaps and recommendations are provided in Table 6. The recommendations were further developed into programmatic requirements that BTRP used to procure relevant services and goods to assist the country with closing identified gaps.

**Table 6.** Examples of Pathogen Consolidation and Security Gaps, Explanations, and Recommendations Generated from a Biological Threat Reduction Program Country Assessment^a^

Gaps	Explanations	Recommendations
Material control and accountability	Unlabeled samples are present in many pathogen repositories. Complete documentation of repository contents is not common nor required by regulations. Paper logbooks are used to track pathogen samples, creating difficulties in maintaining an accurate inventory and chain of custody. Wax seals, an outdated technique, are used to monitor access to pathogens.	Work with key institutions and organizations to develop biosecurity culture. Implement electronic pathogen inventory tool at select facilities, which will improve pathogen inventory and tracking. Develop procedures for chain of custody during sample transport.
Physical security	Security features such as grilles on windows, electronic access control, close-circuit television, volumetric sensors, and integrated alarm systems are broken or not in use in laboratories X, Y, and Z.	Provide security upgrades to selected facilities. Identify mechanisms to support maintenance and sustainment of security features.
Transportation of infectious substances	The absence of national guidance allows transportation of infectious substances via any means available; eg, Agency X supplies packaging material for specific surveillance projects but standardized packaging for emerging infections is not routinely available to laboratories.	Develop protocols for sample collection, packaging, and transport within the country and to international laboratories. Identify and train appropriate personnel in sample handling to ensure specimen viability. Implement sample handling protocols across the Ministry of Health laboratory system.
Pathogen consolidation	Laboratory X is a central reference laboratory and pathogen repository for the country; however, many other laboratories in the country may store pathogens; the number and type of pathogens throughout Country X is unknown by the government.	Develop pathogen consolidation plan to identify vulnerabilities and needs of target facilities. Identify collections of pathogens of security concern at other facilities.
Personnel security	Personnel do not undergo background checks to ensure suitability prior to accessing pathogens of security concern.	Develop system for verifying academic and professional backgrounds of personnel and performing criminal background investigations.

^a^ Gaps and recommendations cover areas that support the Biological Threat Reduction Program and identify where the program can operate due to its specific mandate. Country identifiers were removed to maintain anonymity.

## Discussion and Conclusion

The examination of disease surveillance systems supports prioritization of health security risks and identification of efficiencies where a joint risk management strategy can address mutual risks to public health and veterinary disease surveillance. Clearly, BTRP is not unique in having a defined approach to assessing health security risks; several international processes and frameworks have been developed to understand a country's health security preparedness and response capacity across various technical areas. However, BTRP's health security risk assessment process is distinct in its approach to identifying and reducing risk through a comprehensive in-country evaluation process of national and regional systems and integration of multiple viewpoints and perspectives that serve to increase fidelity and reduce bias.

The BTRP country assessment tool captures not only the perspectives of those conducting the evaluation but also the opinions of in-country partners and stakeholders including academic institutions, third-party experts (US government partners and international organizations), and a full range of SME BSV and BS&S research and analysis. Moreover, the tool can identify health security risk in public health and veterinary systems simultaneously, allowing countries and donors to devise interventional strategies for holistically managing zoonotic events. In addition to stakeholder meetings and evaluations of capacities and capabilities at the national level, the BTRP team also travels to regions within a country to visit facilities and conduct a comprehensive assessment of regional and local human and veterinary systems and One Health approaches. This inclusive approach provides an in-depth evaluation of the risk landscape and gap analysis at the national, regional, and local levels to maximize the scope of the assessment and inform tailored risk mitigation activities throughout the country.

Although the BTRP's 3-phase process allows for each phase to build on and further validate the previous phase, thus minimizing the need for in-country presence during the final stage, the in-country research conducted by the team during the final phase assesses a representative sample to draw, as conclusive as possible, a comprehensive picture of BSV and BS&S capabilities across the country. The standardized approach to data collection, analysis, and reporting—coupled with continuous quality assurance and control—result in high-fidelity data. The identified capability gaps and risks inform the development of BTRP's multiyear country plans and intervention strategies, in close coordination with partner countries, and the analyzed and synthesized information directly informs the development of BTRP project requirements for improving BSV and BS&S capacities in countries, all of which advance global health security.

The country assessment tool identifies biological threats of human and animal origin that may pose global risk through accidental or intentional release. The use of a standardized and comprehensive tool is ideal for identifying effective and sustainable interventions aimed at meeting local and international health security goals to timely and accurately detect, diagnose, and report country relevant pathogens and diseases of security concern. The standardized assessment approach enables BTRP to track progress over time and measure its impact in strengthening public health and veterinary systems around the world, thus contributing to the global effort to mitigate biological threats and, ultimately, reduce the spread of human and animal diseases, decrease loss of human and animal life, and avoid negative economic effects.

## Notes

^*^
For BTRP, biosurveillance is defined as increasing partner nations' capacities to detect, diagnose, and report disease events quickly, regardless of their point of origin (naturally occurring or manmade), and covers human clinical, veterinary, epidemiology, laboratory science, and health information technology domains.

^†^
For BTRP, biosafety is defined as enhancing partner nations' abilities to ensure that all operations involving the handling of pathogens are done in a manner that prevents their accidental release or spread.

^‡^
For BTRP, biosecurity is defined as securing partner nations' pathogen holdings from internal and external threats to ensure those pathogens are not accessed by parties intent on using them for nefarious purposes.

^§^
Select agents and toxins are defined by the US Department of Health and Human Services and the US Department of Agriculture^5^ and identified as especially dangerous due to their abilities to be weaponized by nefarious entities. Select agents and toxins are referred to as especially dangerous pathogens in BTRP.
